# Becoming obese in young adulthood: the role of career-family pathways in the transition to adulthood for men and women

**DOI:** 10.1186/s12889-019-7797-7

**Published:** 2019-11-12

**Authors:** Jarl E. Mooyaart, Aart C. Liefbroer, Francesco C. Billari

**Affiliations:** 1 Department of Sociology, McGill University, Peterson Hall Building 3460 McTavish Street, Montréal, QC H3A 0E6 Canada; 2 Netherlands Interdisciplinary Demographic Institute, Lange Houtstraat 19, 2511 CV The Hague, The Netherlands; 3 Department of Epidemiology, University Medical Centre Groningen (UMCG) / University of Groningen, Groningen, Netherlands; 4 Department of Sociology, VU University Amsterdam, Amsterdam, Netherlands; 5 Department of Social and Political Sciences and Carlo F. Dondena Centre for Research on Social Dynamics and Public Policies, Bocconi University, Via Röntgen 1, 20136 Milan, MI Italy

## Abstract

**Background:**

During the transition to adulthood many young adults become obese for the first time in their lives, yet relatively little research has examined why people in this life phase become obese. This study examines what career and family life-course pathways during the transition to adulthood are related to developing obesity in young adulthood.

**Methods:**

We use data from the NLSY97, a U.S. nationally representative panel survey conducted by the Bureau of Labor Statistics between 1997 to 2013 (*N* = 4688), and apply multichannel sequence analysis in order to identify clusters of typical career-family pathways during the transition to adulthood (age 17 to 27), and subsequently investigate whether these pathways are associated with becoming obese at the end of young adulthood (age 28), using logistic regression. We control for obesity at age 17 and family background factors (race, parental education, parental income, and family structure). To take into account the fact that the transition to adulthood has a different meaning for men and for women, we also interact career-family clusters with gender.

**Results:**

For women, pathways characterized by college education, early home leaving, and postponement of family formation decrease the probability of becoming obese. For men, pathways characterized by early marriage increase the probability of becoming obese.

**Conclusions:**

The results highlight the importance of gender differences in how career and family pathways are related to becoming obese in young adulthood.

## Background

The dramatic increase in obesity over the last few decades in the United States and other Western countries is a major public health concern [12, 32, 36]. Currently, about one in three adults are obese [35]. Because obesity has been linked to an increased risk of a number of diseases (See Kopelman [23] for an overview), it is crucial to identify risk factors for how obesity develops.

While much research has focused on obesity during childhood and adolescence, a large increase in body mass index (BMI) occurs during the transition from adolescence to adulthood [22, 34, 48]. Many youths, having normal weight during their childhood, become obese for the first time during the transition to adulthood [21]. However, the explanation of why such a strong increase in obesity occurs during the transition to adulthood has received little attention [34]. The transition to adulthood is an eventful phase in the life-course. It is the time in which events such as leaving the parental home, entering the labor market, and/or postsecondary education, union formation, and parenthood take place in the lives of most individuals. There is evidence that specific events in the transition to adulthood are related to changes in weight, physical activity and dietary behavior. Events such as leaving the parental home and moving to college are associated with a less healthy diet and a drop in physical activity [57, 59]. Furthermore, getting married, becoming a parent, and starting a new paid job have been related to more unhealthy food intake and a decrease in physical activity [10, 17].

Over the last decades, the transition to adulthood has become destandardized and diversified [46], meaning that there is no longer one typical way in which youths become adults, but rather there are diverse pathways marking the transition to adulthood. Marriage rates have decreased and cohabitation rates have increased [28]. Furthermore, the transition to adulthood has been protracted since the second half of the twentieth century: union formation (be it marriage or cohabitation) and parenthood have been delayed, and youth have prolonger their time spent in full-time education [19]. Given these important changes in the transition to adulthood, it is important to understand which of career and family pathways are nowadays associated with developing obesity in young adulthood.

An important aspect of the transition to adulthood is the adoption of adult roles and responsibilities. The life-course approach acknowledges that individuals do not only move from one role to another; they can also adopt multiple roles at the same time in the career and family domains [17]. The interplay between career and family roles may have an impact on obesity, as the adoption of multiple roles may give rise to a conflict between career and family. Youth who experience family events before the completion of college are more likely to become obese [31]. Work-family conflict is related to more high-fat and high-sugar food consumption and less physical activity and therefore increase in BMI [5, 27, 39, 51]. For men and women both the meaning of career and family roles, and the strategies through which conflicts are managed are different [45]. We therefore can expect that the effect of the transition to adulthood on developing obesity differs by gender. Indeed, there is evidence for a different impact of life-course events and work-family conflict on BMI, diet and physical activity for men and women [5, 24, 39].

Research linking the transition to adulthood with the development of obesity is still limited. While some research focuses on single transitions such as college enrollment [26, 33] and marriage [7, 49, 53], few studies examine the influence of multiple characteristics of the transition to adulthood on BMI. Macmillan and Furstenberg [29] found that employed, married young adults with a 4-year college degree, having become parents after the transition to adulthood show a lower BMI increase than unemployed young adults with no college degree, who have not entered unions or parenthood. Scharoun-Lee et al. [41, 42] found that young adults who become residentially independent and enter the labor market and marriage early have an increased risk of obesity. There is also limited evidence for gender differences in the relationship between the transition to adulthood and obesity. Studies by Scharoun-Lee and colleagues found that for women, being socio-economically disadvantaged throughout the transition to adulthood and foregoing post-secondary education increases the risk of obesity whereas this applies less for men [41–43].

However, these studies do not fully take into account the ordering and timing of both career- and family-related events in the transition to adulthood. Events such as marriage and entering postsecondary education, obtain a specific meaning once the whole pathway of the transition to adulthood is taken into account [2, 6, 16]. While other studies link family and career sequences to health outcomes [11, 40], the present study is the first to link the transition to adulthood as a sequence of events to the development of obesity in young adulthood. Sequences contain information on quantum (which events occur and how many times), ordering (what is the sequencing of events), and timing (when events take place) of events [9]. This approach can provide more insight into what specific life-courses are linked to the risk of becoming obese.

In this study, we focus in detail on the influence of life-course sequences in both career and family domains between ages 17 and 27. In order to deal with career and family sequences simultaneously, we use multichannel sequence analysis [20, 37], which enables us to obtain a measure of similarity between individuals’ career and family sequences. On the basis of these similarity measures, individuals’ career-family sequences are grouped into clusters. In the final step we examine whether membership of a certain career-family sequence cluster is related to a higher or lower probability of developing obesity in young adulthood, with a specific focus on gender. Our research objective is to assess the influence of career-family trajectories on the risk of becoming obese towards the end of young adulthood and whether career-family trajectories are differently related to obesity for men and women. Our main research question is therefore: to what extent are career-family pathways during the transition to adulthood related to becoming obese for men and women?

In assessing differences in the development obesity in young adulthood, also racial and family background differences may play an important role. Black and Hispanic youths are found to have a higher prevalence of obesity compared with whites [35]. Parental SES and family structure are also related to BMI for children from impoverished and broken families and lower-class households, who are more likely to develop obesity during their lifetimes [25, 41, 42, 44, 56, 58]. In the present study, we also take into account the influences of race, parental SES, and family structure, by examining whether these background factors continue to have an influence on becoming obese during young adulthood. The advantages offered by protective factors may accumulate over the life-course, as in the “cumulative advantage” concept [14, 47, 55]. There is indeed evidence that cumulative advantage can also occur with respect to obesity risk [15, 41, 42]. Our research design allows us to test whether certain types of career-family sequences during the transition to adulthood increase the risk of becoming obese in early adulthood, and whether they have an effect independently and on top of disadvantage in childhood.

## Methods

### Data

This study uses data from the National Longitudinal Survey of Youth from 1997 (hereafter referred to as NLSY97), a panel study conducted by the U.S. Bureau of Labor Statistics. Respondents were selected in 1997 at ages 12 to 17 (born 1980–1984), using a multi-stage area (housing units) stratified random sampling design,^1^ and were interviewed annually until 2013 (with the exception of 2012). The NLSY97 contains an oversample of respondents of Afro-American and Latino descent. When weighted, the NLSY97 provides a nationally representative sample. The total sample consists of 8984 respondents. However, we only included those respondents who participated in all waves and for whom there is at least some information on body height and weight at (around) age 28. Most respondents are excluded, because they do not provide full information on the timing of key events in the transition to adulthood, while only few respondents are excluded because of a missing or invalid height and weight. In all, our analysis is based on *N* = 4688 cases (47% men, 53% women). We use sample weights designed specifically for the group of respondents that participated in all waves.

### Obesity definition

The NLSY97 contains measures of self-reported height in feet and inches and weight in pounds (lbs). BMI is calculated by (weight(lbs) × 703)/height^2^(inches). Our main dependent variable is a binary variable indicating whether or not the subject was obese at age 28, with this age chosen also because all respondents in the survey were at least 28 years old. If respondents did not report height and weight at age 28, their BMI at age 29 was used, and if this was also missing, their BMI at age 27 was used. In line with common practice [13], respondents were classified as obese when their BMI was 30 or higher. Furthermore, adopting the same approach as MacMillan and Furstenberg [29], all BMI scores below 12 or over 50 were considered invalid.

### Family background and control variables

The first NLSY97 wave contains a “Parent Questionnaire”, from which we derived family background characteristics, such as parental income, education, and family structure. *Parental education* was coded as the highest education of the mother or father using five categories: lower than high school, high school, some college, 4-year college or higher, and missing. *Parental income* refers to the household income reported by one of the parents when the respondent was 12 to 16 years old and was coded in quartiles, also including a missing category. *Family structure* is the recorded family structure in 1997 and was coded in four categories: 1) Both biological parents, 2) 1 biological, 1 step-parent, 3) 1 biological parent, 4) other (no biological parents). For the main respondent, the gender variable (*Female*) was coded 0 for males and 1 for females, and *Race* was coded in four categories: 1) white (non-Hispanic), 2) black (non-Hispanic), 3) Hispanic, 4) other (mixed). Finally, two controls were included. First, we control for obesity at the end of adolescence, so that we can examine how career-family sequences during the transition to adulthood affect the probability of becoming obese, rather than possibly viceversa obesity affecting career-family trajectories. We therefore included the variable *Obesity age 17* as a dichotomous variable (0 = not obese, 1 = obese). We defined obesity at age 17 at a cut-off point of 28 rather than 30, as previous research has shown that a somewhat lower cut-off point more accurately captures obesity at younger ages [38]. Second, *pregnant* indicates whether the respondent was pregnant (1) or not (0) at age 28.

Table 1 shows the proportions of all the categories of the family background variables in the sample and the percentage of obesity within these categories.

**Table 1:** Descriptive statistics on family background variables (*N* = 4688)

	Proportion in sample (%)	Obesity at Age 17 (%)	Obesity at Age 28 (%)
Gender			
Male	47.11	15.58	31.26
Female	52.89	14.12	34.27
Parental income			
Quartile 1	18.79	19.93	40.54
Quartile 2	18.91	17.55	35.66
Quartile 3	19.23	15.04	34.51
Quartile 4	19.77	8.29	23.90
Missing	23.30	13.79	30.59
Parental education			
Less than high school	15.32	20.00	40.42
High school diploma	31.06	16.10	36.03
Some college	23.79	16.19	33.36
4 year college or more	25.45	9.03	23.58
Missing	4.38	13.59	34.95
Family structure			
Both biological parents	52.15	12.28	30.27
1 biological, 1 step parent	12.26	14.06	30.21
Single parent	30.53	18.75	37.56
Other	5.06	18.91	37.39
Race			
White	52.49	11.19	27.20
Black	26.40	19.34	41.34
Hispanic	20.11	17.88	36.08
Other	1.00	23.40	40.43

### Analytical strategy

#### Multichannel analysis of career-family sequences

In NLSY97, respondents reported the year and month in which specific life-course events occurred. In terms of education, in each wave they were asked whether they had entered or exited an educational institution in the previous year. Respondents were also asked to report the level of education in which they enrolled, i.e., secondary school, 2-year college, or 4-year college (including postgraduates). Regarding employment, respondents were asked to provide the start and end dates of each job they had in the previous year, including the number of working hours.^2^ With respect to family formation characteristics, respondents were asked whether they had started or ended a marriage or cohabiting relationship in the previous year, as well as the year and month of birth of each of their children. In each wave, respondents reported who was living in their household at that time. Furthermore, respondents were asked the month and year in which they first left and returned to the parental home (if they had done this).^3^

We use NLSY97 information to construct a sequence-type life-course dataset, creating, for each individual, a sequence of 96 consecutive months between ages 17 and 27, along two dimensions: career and family. In order to create a sequence dataset it is necessary to define the ‘state space’, consisting of the different states individuals can occupy at each time-point. The career states cover educational enrollment and employment status. Respondents are classified as being enrolled in high school, in a 2-year college education, a 4-year college education, or not enrolled. Where there are gaps between educational episodes, we consider someone as continuously enrolled if those gaps are shorter than 3 months. Regarding employment, individuals are classified as employed 35 h per week or more, employed for less than 35 h per week, or not employed (the last category includes people who are not actively seeking employment, for instance stay-at-home mothers). Combining these educational and employment statuses leads to 12 (4 × 3) possible different career states.

Family states are defined in terms of living arrangements and parenthood status. Four living arrangements are distinguished: living with parents, living alone/independent, living with partner (cohabiting), and living with spouse (marriage). Within each of these options the respondent can either have had a child or not. Entering parenthood is considered irreversible. Once respondents have become parents, they are classified as parents for the rest of the sequence, independently of whether they co-reside with the child. This leads to 8 (4 × 2) possible family states.

Multichannel sequence analysis has been developed to compare life-course sequences on multiple dimensions [20, 37], such as career and family. In multichannel sequence analysis, sequences are compared on both dimensions simultaneously. The pathways of two different individuals are similar if the timing, occurrence, ordering, and duration in states are similar to each other in both the career and family sequences. In order to develop a series of ideal-typical pathways in the transition to adulthood, we start from a dissimilarity, or distance, matrix and use cluster analysis. We use Optimal Matching Analysis to measure the level of dissimilarity of sequences [1]. The measure is based on how many states would have to be substituted, deleted, or inserted in order to transform one sequence into another. The more of these operations are required, the less similar the sequences are. However, some life-course transitions may occur more often than others. Therefore, in line with the literature we assign costs of substitutions based on the transition rates between different states [52]. When the transition rate from one state to another is low, the substitution costs for these states is high, leading therefore to a larger distance between sequences.

Multichannel sequence analysis is performed using the TraMineR package in R. Based on the distance matrix resulting from the multichannel Optimal Matching procedure, a weighted (using NLSY97 weights) hierarchical clustering procedure using Ward’s method was chosen to produce clusters of respondents with similar life sequences. An advantage of the Ward algorithm is that it produces fairly equal-sized groups [3].

The choice of the optimal number of clusters is based on the best model fit in terms of the Akaike Information Criteria (AIC) [4]. We conduct multiple logistic regressions, in which each logistic regression differs in the number career-family clusters (based on the different cluster solutions) that are included as dummy variables, in order to test which set of career-family pathway variables most adequately predict obesity at age 28. Table 2 shows that the 8-cluster solution provides the lowest AIC and therefore the best model fit, thus we opt for the 8-cluster solution. .

**Table 2:** Model fit (AIC) of logistic regression for different number career-family clusters

Number of clusters	AIC
4	4887.19
5	4880.47
6	4883.93
7	4878.49
8	4876.91
9	4877.81
10	4881.52

#### Analyzing precursors of obesity

We use binary logistic regression to identify the effects of career-family sequences on the risk of obesity at age 28. In addition to the family background and control variables, dummy variables for the set of career-family sequence clusters during the transition to adulthood are included, indicating whether someone is a member of a particular career-family cluster. The career-family cluster variables are interacted with gender in order to examine differences in the influence of each career-family type between men and women. Weights constructed by the NLSY were used to counter any potential selectivity of the sample.

## Results

### Descriptive results on the transition to adulthood

**Fig. 1:**
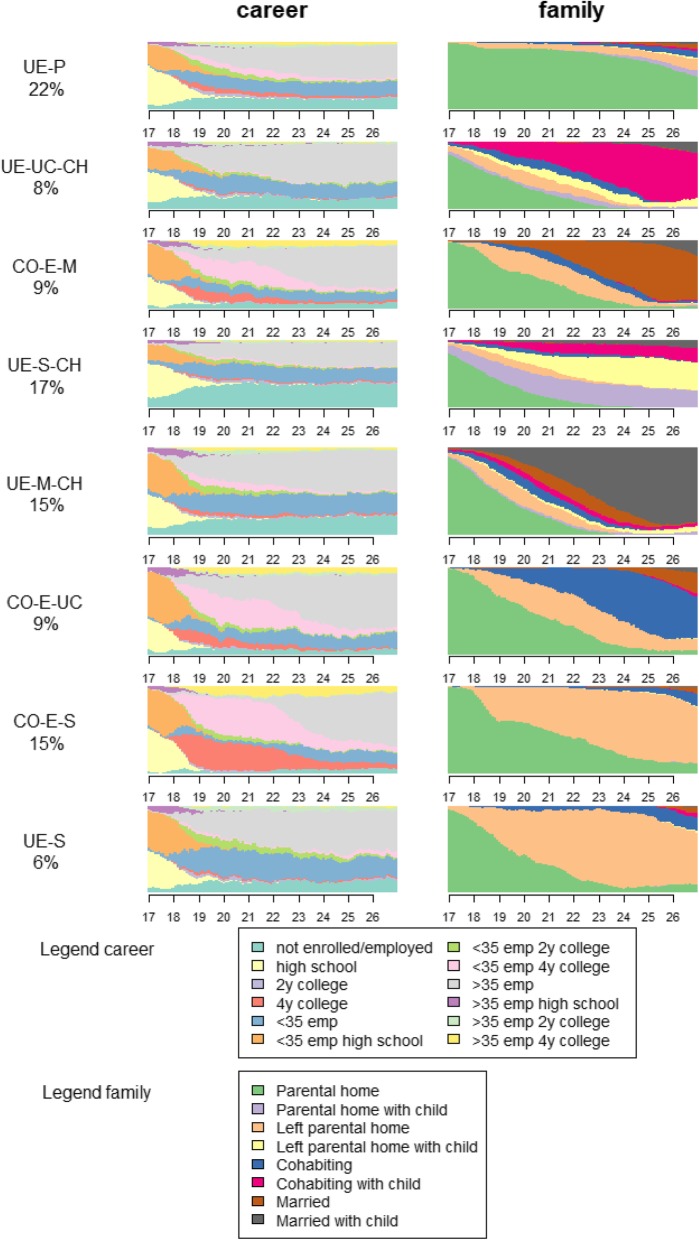
Distribution of states for each of the multichannel career-family sequence clusters

In Fig. 1 we describe the eight career and family clusters. Some clusters have a similar career sequence, but differ in their family sequence and vice versa. To label the clusters we use a coding system that highlights whether most individuals in the cluster attend college (CO), are continuously employed (E) or have more unstable employment (UE). For what concerns family behavior, our labels use the main relationship/residential status: married (M), unmarried cohabitation (UC), single living (S) or in parental home (P), and lastly whether the majority of individuals has a child (CH). In the first cluster, the majority of young adults spend most of their time in the parental home. Regarding career pathways, respondents in this cluster spend little time in college and most end up in full-time employment, followed by part-time employment, and then inactivity. We therefore label this cluster UE-P. In the second cluster, the vast majority cohabit and have a child. Almost no one in this cluster attends college and employment is relatively unstable, giving this cluster the UE-UC-CH code. The third cluster we label CO-E-M. Almost all respondents in this cluster are married, but relatively few have had children. Most spend time in either 2- or 4-year college education. The vast majority have stable full-time employment. The fourth cluster includes respondents who (previously) entered cohabitation or marriage, but by age 27 the majority have had a child and are not in a cohabiting relationship. Of all the clusters, respondents in this one spend most of their time in inactivity and least in employment and hardly anyone attends college. Therefore, we label this cluster UE-S-CH. In the fifth cluster, respondents marry and have children in quick succession. Most people in this cluster are in employment, either full-time or part-time at age 27, but there is also quite some time spent in inactivity, and few enter college, hence the label UE-M-CH. Entering cohabitation but not having children is the most salient feature of the sixth cluster. Most remain in cohabitation although some marry or become single again. Most enter college and have full-time employment when they reach 27. The label for this cluster is CO-E-UC. In the seventh cluster, almost all attend a 4-year college education. At age 27 most have finished their college education and have entered full-time employment. Regarding the family pathways of this group, most have left the parental home but experienced no other events, hence the label CO-E-S. In the final cluster, respondents spend very little to no time in college education. Most are full-time employed at age 27, but there is also time spent in part-time work and inactivity. They leave the parental home, but do not enter a union or have a child, thus the label for this cluster is UE-S.

Table 3 shows the composition of the eight career-family clusters in terms of obesity at ages 17 and 28, gender, and pregnancy status at age 28. The UE-P cluster has the highest percentage of obesity at age 17 (20.56%), and the CO-E-M the lowest with 7.43%. At age 28, the cluster with the highest percentage of obesity is US-S-CH (38.22%), with the cluster CO-E-S having the lowest (21.25%). Pregnancy at age 28 is most prevalent in the CO-E-M cluster (18.47%), and least prevalent in the UE-S cluster (4.47%).

**Table 3:** Obesity, gender, pregnancy and career-family sequence cluster membership (%)

	UE-P	UE-UC-CH	CO-E-M	UE-S-CH	UE-M-CH	CO-E-UC	CO-E-S	UE-S
Obesity at 17								
No	79.44	82.67	92.57	81.94	86.94	90.02	91.01	83.51
Yes	20.56	17.33	7.43	18.06	13.06	9.98	8.99	16.49
Obesity at 28								
No	61.97	63.07	68.82	61.78	64.61	75.18	78.75	67.35
Yes	38.03	36.93	31.18	38.22	35.39	24.82	21.25	32.65
Gender								
Men	62.56	45.74	40.53	31.68	36.10	38.69	53.95	68.04
Women	37.44	54.26	59.47	68.32	63.90	61.31	46.05	31.96
Pregnant at 28								
No	95.33	91.19	81.53	86.78	86.38	88.56	94.41	95.53
Yes	4.67	8.81	18.47	13.22	13.62	11.44	5.59	4.47

The career-family clusters are coded using a the following scheme: *CO* college education, *E* (stable) employed, *UE* unemployed or unstable employment, *M* married, *UC* unmarried cohabitation, *S* single, P = living in the parental home, CH = having (had) a child(ren)

Table 4 displays the distribution of career-family pathways cluster membership for the different family background variables. The respondents in the CO-E-S and CO-E-UC clusters appear to be generally from more advantaged background as they predominantly white, have on average higher educated parent, grew up in relatively richer households, with a higher share of two-parent families. Respondents in the UE-S-CH and UE-UC-CH clusters appear to be on average from a more disadvantaged background, with a higher share of racial minorities, with less affluent and educated parents, and a lower share of two-parent families. However, even though there are differences between the clusters in terms of family background, all clusters have a minimum of 5% per category (with the exception of “missing” or “other” categories, which have lower percentages among all different career-family pathway clusters), so that there is representation of all family backgrounds in each of the clusters.

**Table 4:** Family background and career-family sequence cluster membership

	UE-P	UE-UC-CH	CO-E-M	UE-S-CH	UE-M-CH	CO-E-UC	CO-E-S	UE-S
Parental education								
< High school	18.37	21.31	8.39	25.39	19.66	7.79	4.22	8.59
High school	32.57	42.90	23.26	41.75	31.18	29.93	17.03	31.96
Some college	27.21	22.44	24.94	19.76	24.86	23.84	19.62	29.90
4-year col.	17.48	7.95	39.57	9.29	17.70	35.04	55.59	26.12
Missing	4.37	5.40	3.84	3.80	6.60	3.41	3.54	3.44
Parental income								
Quartile 1	19.46	26.42	7.91	33.12	20.22	11.68	8.86	16.15
Quartile 2	20.16	25.85	16.55	22.64	19.24	18.00	10.35	21.99
Quartile 3	18.57	19.03	24.70	12.57	21.63	23.84	18.80	19.93
Quartile 4	16.48	8.52	30.22	6.02	14.89	25.79	38.42	22.34
Missing	25.32	20.17	20.62	25.65	24.02	20.68	23.57	19.59
Race								
White	43.79	36.93	74.82	25.52	57.3	73.72	68.80	57.39
Black	29.89	32.10	8.15	56.81	13.76	10.71	20.16	22.68
Hispanic	25.32	29.55	15.83	16.88	28.23	14.84	9.95	18.21
Other	0.99	1.42	1.20	0.79	0.70	0.73	1.09	1.72
Family structure								
Both parents	56.21	38.35	66.43	28.53	54.35	59.37	66.21	45.70
1 bio 1 step	9.33	17.05	12.71	13.74	13.62	14.84	9.13	12.71
Single parent	30.09	36.93	17.75	48.43	26.83	24.33	21.80	35.05
Other	4.37	7.67	3.12	9.29	5.20	1.46	2.86	6.53

The career-family clusters are coded using a the following scheme: *CO* college education, *E* (stable) employed, *UE* unemployed or unstable employment, *M* married, *UC* unmarried cohabitation, *S* single, P = living in the parental home, CH = having (had) a child(ren)

### Logistic regression results

Results of a binary logistic regression model, with obesity risk at age 28 as the dependent variable, are presented in Table 5. The analyses in Table 5 include the interaction between gender, and the career-family pathway cluster dummies (results without the interaction are available upon request). Noticeable is the strong effect of obesity at age 17. Respondents who were obese at age 17 are more than 16 times more likely to be obese at age 28 compared with those who were not obese at age 17. Two significant family background effects are observed. First, young adults who have one or more university educated parents have a lower risk of being obese at age 28 compared to those whose parents do not have more than a high school education. Second, blacks have an increased probability of being obese at age 28 compared with whites. There are no significant effects for parental income and family structure.

**Table 5:** Log-odds estimates (and SE) from a logistic regression model with obesity risk at age 28 as the dependent variable

	Coefficient	Standard error
Constant	−1.020^***^	0.191
Obesity age 17	2.798^***^	0.126
Female	0.367^*^	0.181
Pregnant at 28	0.373^**^	0.135
Parental income		
Quartile 1	ref.	
Quartile 2	−0.105	0.130
Quartile 3	0.053	0.138
Quartile 4	−0.269	0.151
Missing	−0.233	0.129
Parental education		
Less than high school	ref.	
High school diploma	−0.091	0.132
Some college	−0.275	0.143
4 year college or more	−0.436^**^	0.152
Missing	0.028	0.224
Family structure		
Both biological parents	ref.	
1 biological, 1 step-parent	−0.126	0.131
Single parent	0.025	0.101
Other	−0.237	0.204
Race		
White	ref.	
Black	0.367^***^	0.103
Hispanic	0.039	0.112
Other	0.326	0.333
Career-family clusters		
UE-P	ref.	
UE-UC-CH	−0.085	0.237
CO-E-M	0.470^*^	0.214
UE-S-CH	−0.198	0.208
UE-M-CH	0.295	0.190
CO-E-UC	−0.180	0.236
CO-E-S	−0.176	0.177
UE-S	−0.035	0.214
Interactions Career-family clusters*female		
UE-P*female	ref.	
UE-UC-CH*female	−0.008	0.347
CO-E-M*female	−0.659^*^	0.300
UE-S-CH*female	−0.031	0.277
UE-M-CH*female	−0.532^*^	0.268
CO-E-UC*female	−0.297	0.326
CO-E-S*female	−0.772^**^	0.276
UE-S *female	−0.220	0.379
Observations	4688	

Obesity is the dependent variable and defined in the model as a dichotomous variable indicating 0 = not obese 1 = obese at age 28, this coding applies also for the independent variable obesity at age 17

The career-family clusters are coded using a the following scheme: *CO* college education, *E* (stable) employed, *UE* unemployed or unstable employment, *M* married, *UC* unmarried cohabitation, *S* single, P = living in the parental home, CH = having (had) a child(ren)

*p < 0.05, **p < 0.01, ***p < 0.001

From Table 5 we learn that there are significant differences between some career-family clusters, and that these differences are gendered. Because of the interaction with gender, the coefficients under “career-family clusters” represent the effects for men. The reference category for the career family clusters variable in Table 5 is UE-P, i.e. those with unstable employment while still living in the parental home. All coefficients in Table 5 therefore indicate the relative difference with respect to those in the UE-P cluster. Since not all relative differences can be shown in the table, we ran the same analysis with different reference categories in order to reveal all significant differences between each pair of career-family clusters (results available upon request). There is a clear positive effect for the CO-E-M cluster, i.e. those who went to college and are stably employed and married with no children, showing a higher risk of obesity at age 28 for this cluster compared with men in the UE-P, UE-UC-CH, UE-S-CH, CO-E-UC, and CO-E-S clusters. Men who are married and have children, i.e. those in the UE-M-CH cluster, have a significantly higher risk of obesity compared with the UE-S-CH and CO-E-S clusters. All other differences between clusters for men are not statistically significant.

The interaction terms show how the cluster effects of women differ from those of men. The negative and significant effects for CO-E-M and UE-M-CH are similar in size to the positive main effect (for men), meaning that for women, being in these clusters is not related to a higher probability of obesity at age 28 with respect to the reference category (UE-P). The interaction with the CO-E-S cluster also shows a negative effect. However, because the effect for men was already negative, this indicates that for women there is a strong negative effect of being in the CO-E-S cluster. Thus, particularly women who attend college, have stable employment afterwards and remain single have a lower risk of becoming obese. In fact, women in this cluster have a lower risk of becoming obese than all other groups of women. The only other statistically significant difference between career-family clusters among women is that those in the CO-E-UC cluster have a lower obesity risk at age 28 compared to those in the UE-P cluster.

**Fig. 2:**
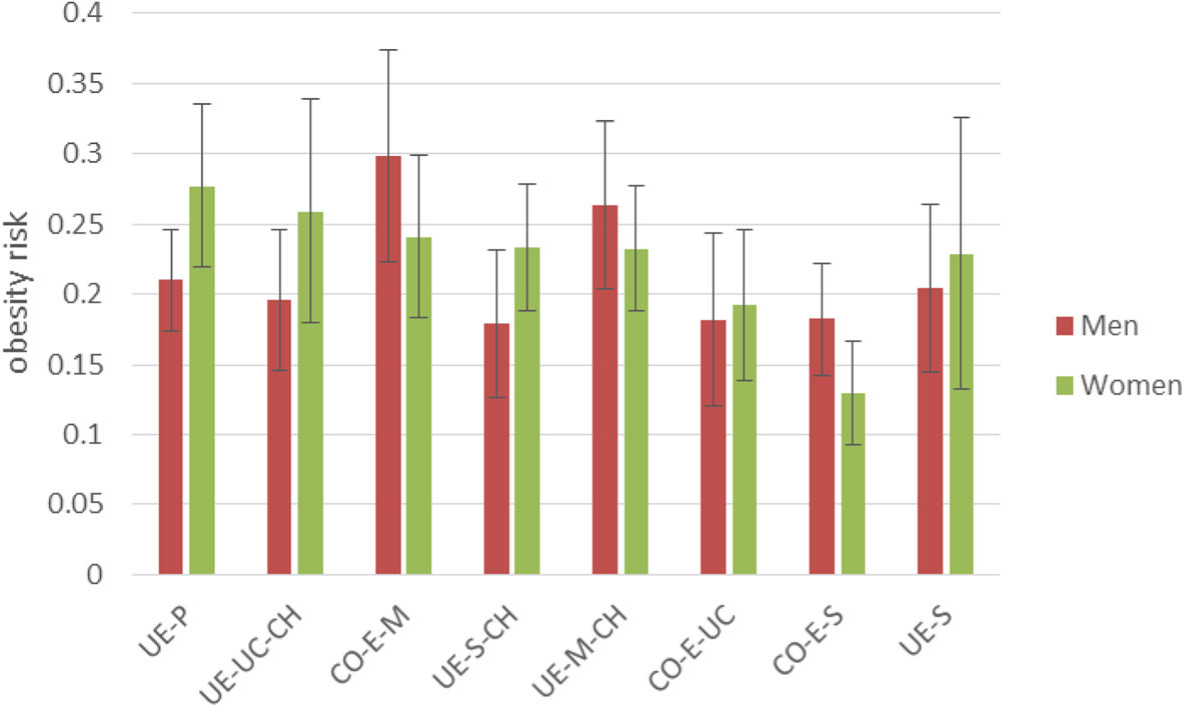
Predicted probability of obesity for each career-family cluster, split by gender, for those who are not obese at age 17

In order to facilitate the interpretation of results, in Fig. 2 we show the predicted probability of obesity at age 28 for those who were not obese at age 17, for each of the career-family clusters, split by gender.^4^ We report the predicted probability of obesity for respondents who were not obese at age 17, because we want to focus on which of the different career-family clusters are related to becoming, rather than to staying, obese. Figure 2 shows substantial gender variation within some of the clusters. Men who are in the CO-E-M cluster have the highest probability of becoming obese (30%). Among men, those following a UE-M-CH type of sequence have a 26% probability. The lowest probability, around 18%, is for men in the UE-S-CH, CO-E-S, and CO-E-UC clusters. Men in other clusters have around a 20% probability of becoming obese.

For women, the ordering of career-family clusters in terms of highest to lowest probability of becoming obese is very different from that of men. Women in the UE-P cluster have a 28% probability of becoming obese and thereby have the highest risk among women. Next, the UE-UC-CH cluster has a 26% probability. At the lower end in terms of obesity risk are women in the CO-E-M cluster (19% probability), but the lowest obesity risk of all is found for women in the CO-E-S cluster (13% probability). Women in the other career-family clusters have around a 23–24% probability of becoming obese.

## Discussion

In line with previous studies, we found that obesity in adolescence is strongly related to obesity in adulthood [22, 34, 48]. While generally those who attend college and postpone childbearing have lower obesity risk, an important finding of this study is that career-family pathways during the transition to adulthood have different associations with the risks of becoming obese for men and for women.

Women who typically attend 4-year college education, leave the parental home in their early 20s, but postpone union formation and parenthood, have a much lower risk of becoming obese at age 28 compared to women following other career-family pathways. However, this is not merely because of the postponing effect of education on family formation. Women who postpone family formation and forego any postsecondary education, have a significantly higher risk of developing obesity than their peers who follow the same family pathways but do attend college. Generally, women who experienced early childbearing had a higher risk of becoming obese, compared to those in clusters in which no childbearing took place before age 28. This is in line with the idea that work-family conflict can increase BMI [5, 51]. Yet, somewhat surprisingly, women who stayed in the parental home had the highest risk of developing obesity. It may be that this group of women share particular features that remain unobserved in our analyses. A potential reason could be that staying in the parental home relatively long compared with their peers increases their level of stress. Women who make off-time delayed transitions report higher levels of stress compared to those going through transition such as leaving the parental home and entering marriage at more normative ages [8]. Stress has been linked to obesity, as it can lead to an unhealthy diet [50].

For men, the picture is quite different. Early marriage seems to be the defining characteristic of increased obesity risk. Surprisingly, men who marry early but do not have a child appear to have the highest risk of developing obesity. A possible explanation for the increase in BMI after marriage is that those who are still in the ‘marriage market’ may be more keen to maintain a healthy body weight in order to attract a potential marriage partner [7, 54]. However, one would expect than expect that those marrying early and having one or more children would also have the same obesity risk, or perhaps higher given the higher family burden, but this is not corroborated by our analyses. Furthermore, results show that those who marry and have children early most often do not enter college, whereas men who marry early but do not have children (yet) often do attend college. Thus, it appears that college education does not buffer the risk of becoming obese among men that marry early. However, one would expect this equally strong for both genders which appears not to be the case. Perhaps, women who are married with children improve their diet compared with women who are married without children. Lake et al. [24] found that women often change their diet after childbirth and that within cohabitation and marriage they exert a positive influence on the diet of their partner, more so than men do on their female partners.

In addition to the impact of career-family pathways during the transition to adulthood, we find some family background effects. We find a decreased risk of becoming obese for those with at least one parent with a 4-year college degree or more compared with those whose parents have no more than a high school degree. This suggests that there is cumulative advantage on the basis of education, as the advantage of a decreased risk of developing obesity by following a “4-year college” sequence and having highly educated parents stack up. Furthermore, we find that blacks compared with whites have a higher risk of becoming obese in young adulthood. The reason we do not find other effects of family background could be that these effects are mediated through the career-family sequences in the transition to adulthood and obesity at adolescence.

This study has some limitations. First, BMI was calculated based on self-reported height and weight. There is evidence indicating a small bias in these self-reports because height tends to be over-reported and weight overestimated by men, while underestimated by women [30]. Second, this study has shown that career-family sequences in the transition to adulthood are related to the risk of becoming obese, but it has not revealed the exact mechanisms by which these pathways impact the risk of obesity. Future research should therefore examine more specifically the mechanisms, for instance through change in diet and physical activity, by which life-course transitions and role combinations and obesity are related.

## Conclusion

This study has shown that different career-family pathways are related to different risks for developing obesity during young adulthood. Furthermore, results also show that there is a clear gender component in this relationship. For women, a combination of college education and the postponement of family formation clearly buffer elevated obesity risks. Women who have a family to take care of, next to having a job in their early 20s, have a higher risk of developing obesity. Helping women deal with work-family conflict, through better family-oriented work policies may therefore lower their risk of becoming obese. However, women who stay relatively long in the parental home and do not attend postsecondary education have the highest risk of becoming obese, which may be related to the stress of having the feeling of “lagging behind” in terms of the transition to adulthood with respect to their peers. Still, more research is needed to understand why this group in particular have a higher risk of becoming obese. For men, attending college education lowers the risk to become obese, but not when college education is combined with early marriage.

Our results provide two general policy implications in battling obesity in young adulthood. First, not only single factors or events in the transition to adulthood matter. It is fundamental to consider the obesity-related risks of combinations of events and states over the life-course which are related to becoming obese in young adulthood, also through a gendered lens. Second, policy makers should be aware that helping young adults change their life-course pathways can be beneficial in reducing obesity in young adulthood, with for instance helping individuals who struggle in the transition to adulthood to leave the parental home, get a full-time job or attend education. This life-course perspective may not only be helpful in informing policy on how to reduce obesity, but can also be useful in reducing other health risks over the life-course.

## Data Availability

This research uses open access data. For more information go to https://www.nlsinfo.org/content/cohorts/nlsy97 The data can be downloaded from the following repository after making an account: https://www.nlsinfo.org/investigator/pages/login.jsp

## Abbreviations

AIC: Akaike Information Criteria
BMI: Body Mass Index
NLSY97: National Longitudinal Survey of Youth 1997

## Footnotes

1 More information on the sampling design can be found on: https://www.nlsinfo.org/content/cohorts/nlsy97/intro-to-the-sample/sample-design-screening-process

2 The NLSY97 reports weekly job status. We recoded this to monthly statuses using the conversion recommended by the NLS. If someone is employed for at least one week during that period, this person is considered employed.

3 These questions were included from 2003 onwards, but in 2003 respondents also indicated the month and year of home return if this occurred in any of the previous years.

4 Pregnant women at age 28 were excluded
